# Supplementary material for: Missense mutation of Fmr1 results in impaired AMPAR-mediated plasticity and socio-cognitive deficits in mice
Source: Nat Commun. 2021 Mar 10;12:1557. doi: 10.1038/s41467-021-21820-1 (PMC7946954; doi:10.1038/s41467-021-21820-1)
Supplement: Supplementary file 1 — Supplementary Information [file 41467_2021_21820_MOESM1_ESM.pdf]

Supplementary information

Abnormal AMPAR-mediated synaptic plasticity, cognitive and autistic-like behaviors in a missense *Fmr1* mutant mouse model

Prieto, Folci et al.

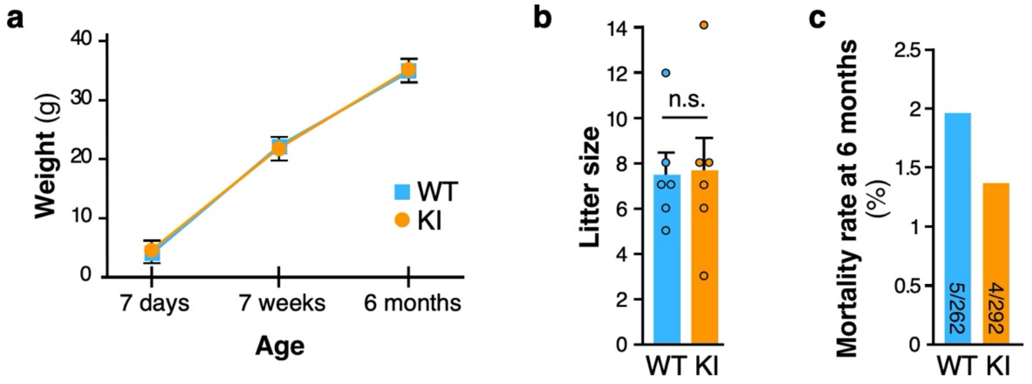

Supplementary figure 1

Supplementary figure 1: *Fmr1^{R138Q}* mice does not show gross alterations. Quantification shows no significant differences between genotypes in the weight **(a**; N=5 animals per genotype**)** of infant, adolescent and adult WT and *Fmr1^{R138Q}* KI mice, their fertility index **(b**; N=6**)** and mortality rate at 6 months **(c)**. The number of deaths is indicated on the bars **(c)**. Data in **a** and **b** are presented as mean values ± s.e.m. Unpaired t-test. n.s., not significant. Source data are provided as a Source Data file.

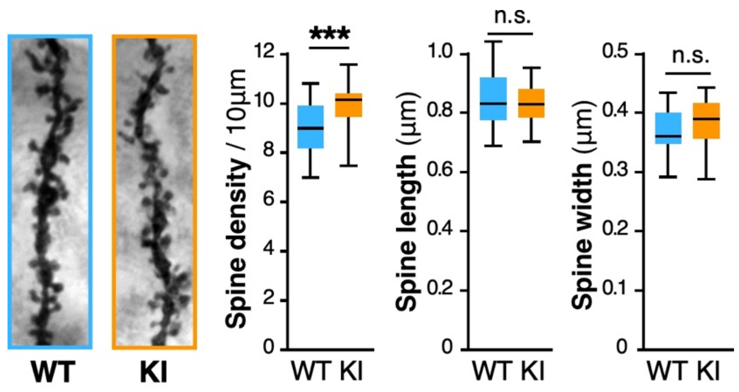

Supplementary figure 2

Supplementary figure 2: The *Fmr1^{R138Q}* hippocampus exhibits increased dendritic spine density. Images of Golgi-stained apical secondary dendrites of CA1 hippocampal neurons from PND90 WT and *Fmr1^{R138Q}* KI littermates. Box plots indicate median (middle line), 25th, 75th percentile (box) and min to max values (whiskers) obtained for the density of spines, spine length and width from WT and *Fmr1^{R138Q}* CA1 secondary dendrites. N = 30 neurons per genotype from 3 independent experiments (1500-2000 spines analyzed per genotype). Two-tailed Mann-Whitney test; n.s., not significant. ***p=0.0002. Source data are provided as a Source Data file.

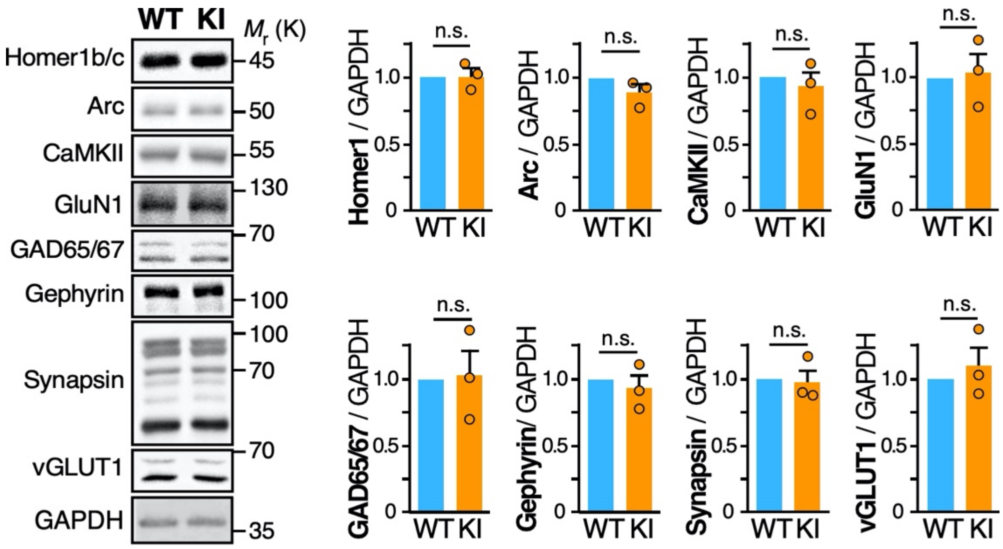

Supplementary figure 3

Supplementary figure 3: Steady state levels of the indicated synaptic proteins in *Fmr1*^{R138Q} brain. Immunoblots showing the levels of the indicated synaptic proteins in brain homogenates from WT and *Fmr1*^{R138Q} KI mice. GAPDH was used as a loading control as in figure 3a. Quantification shows the mean values ± s.e.m. of the total levels of the indicated proteins. N = 3 biologically independent samples. Two-tailed ratio t-test. n.s., not significant. Source data are provided as a Source Data file.

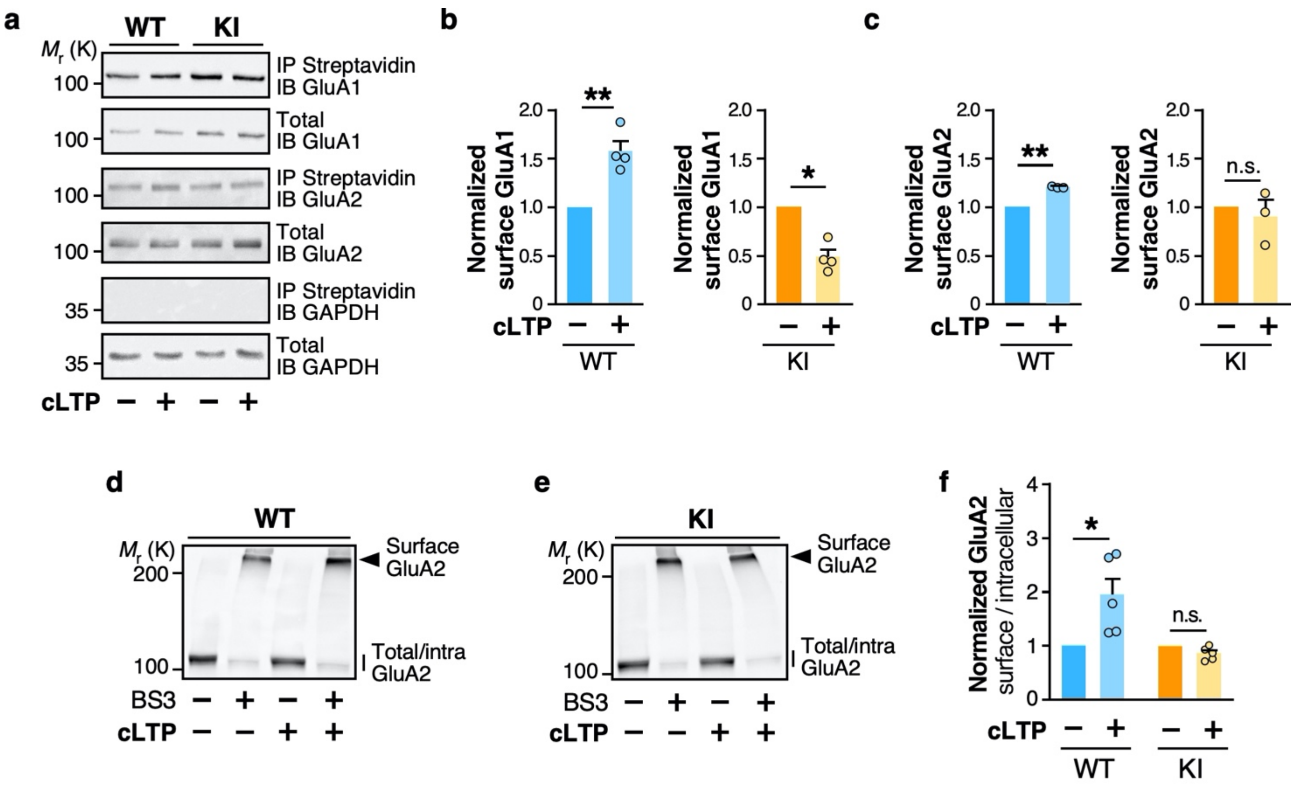

Supplementary figure 4

Supplementary figure 4: Surface GluA2 levels are not increased in cLTP-induced *Fmr1^{R138Q}* hippocampal cultures and slices. a Immunoblots showing the surface expression of GluA1 and GluA2 in basal conditions and upon cLTP induction using biotinylation assays in cultured TTX-treated WT and *Fmr1^{R138Q}* hippocampal neurons. Histograms show the mean ± s.e.m. of the surface levels of GluA1 **(b)** and GluA2 **(c)** from experiments in **(a)**. N=4 (GluA1) and N=3 (GluA2) independent experiments. Two-tailed ratio t-test. n.s., not significant. **b** **p=0.0062, *p=0.0139; **c** **p=0.0012. **d,e** Immunoblots showing the surface expression of GluA2 in basal and cLTP-induced conditions in TTX-treated WT **(d)** and *Fmr1^{R138Q}* **(e)** hippocampal slices using BS3 crosslinking assays. **f** The surface/intracellular ratio in the WT was set to 1 and *Fmr1^{R138Q}* KI values were calculated respective to the WT. Bars show the mean ± s.e.m. N = 5 biologically independent experiments. Two-tailed ratio t-test. *p=0.039. n.s., not significant. Source data are provided as a Source Data file.

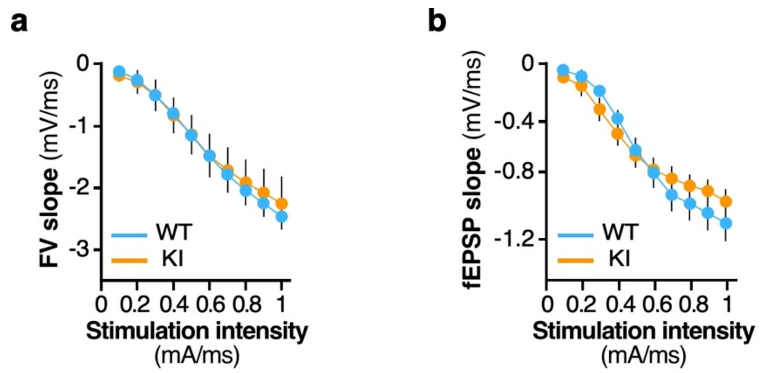

Supplementary figure 5

Supplementary figure 5: *Fmr1^{R138Q}* mice show normal CA3 to CA1 synaptic connectivity. a-b Input/output curves of Fiber Volley (FV, **a**) and postsynaptic responses (fEPSP, **b**) following Schaffer collaterals stimulations in WT and *Fmr1^{R138Q}* hippocampal slices. N = 4 independent experiments. Error bars show the s.e.m. Source data are provided as a Source Data file.

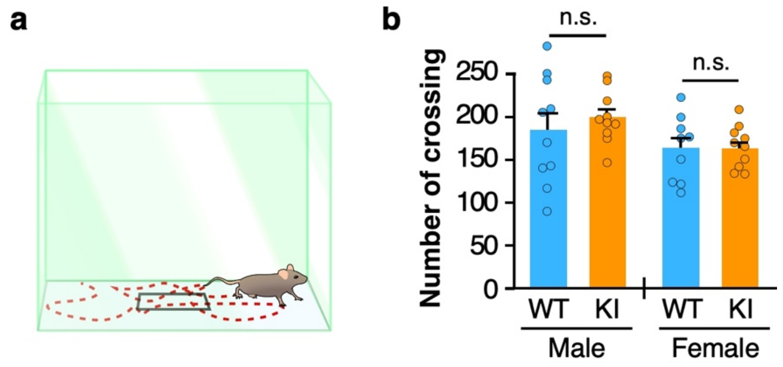

Supplementary figure 6

Supplementary figure 6: *Fmr1^{R138Q}* mice do not show impaired locomotion. a Scheme of the open field test used to assess locomotion. **b** Quantification shows no significant differences between genotypes and genders. Data are presented as mean values ± s.e.m. WT, N = 10 males, 9 females; KI *Fmr1^{R138Q}*, N = 10 males, 10 females. Two-way ANOVA with genotype and sex as factors followed by Newman-Keuls post-hoc test for individual group comparisons. n.s., not significant. Source data are provided as a Source Data file.

Supplementary Table 1: Oligonucleotides forward (F) and reverse (R) used in RNA work.

*Fmr1*_F	GAACAAAAGACAGCATCGCT	*Fmr1*_R	CCAATTTGTCGCAACTGCTC
*Map1b*_F	TCCGATCGTGGGACACAAACCTG	*Map1b*_R	AGCACCAGCAGTTTATGGCGGG
*PSD95*_F	GGCGGAGAGGAACTTGTCC	*PSD95*_R	AGAATTGGCCTTGAGGGAGGA
*Gria1*_F	GAGGTCCCGTAAACCTAGCG	*Gria1*_R	CGGAGTCCTTGCTTCCACAT
*Fxr1*_F	GTGCAGGGTCCCGAGGT	*Fxr1*_R	GGTGGTGGTAATCGGACTTC
*Kif3c*_F	GGTCCCATCCCAGATACAGA	*Kif3c*_R	CCAGAAAGCTGTCAAACCTC
*Pp2a*_F	GTCAAGAGCCTCTGCGAGAA	*PP2a*_R	GCCCATGTACATCTCCACAC
*Homer1*_F	ACCATGTAACCCCGGCTG	*Homer1*_R	CCTTGATGTCAGGATCCCC
*Grm5*_F	GGTCTCTCTCCAGCCATGAG	*Grm5*_R	AGACTTCTCGGATGCTTGGA
